# Supplementary material for: The spindle assembly checkpoint and speciation
Source: PeerJ. 2020 May 11;8:e9073. doi: 10.7717/peerj.9073 (PMC7224227; doi:10.7717/peerj.9073)
Supplement: Supplemental Information 1 [file peerj-08-9073-s001.doc]

**SUPPLEMENTARY MATERIAL FOR**

**The Spindle Assembly Checkpoint and Speciation**

Robert C. Jackson and Hitesh B. Mistry

**
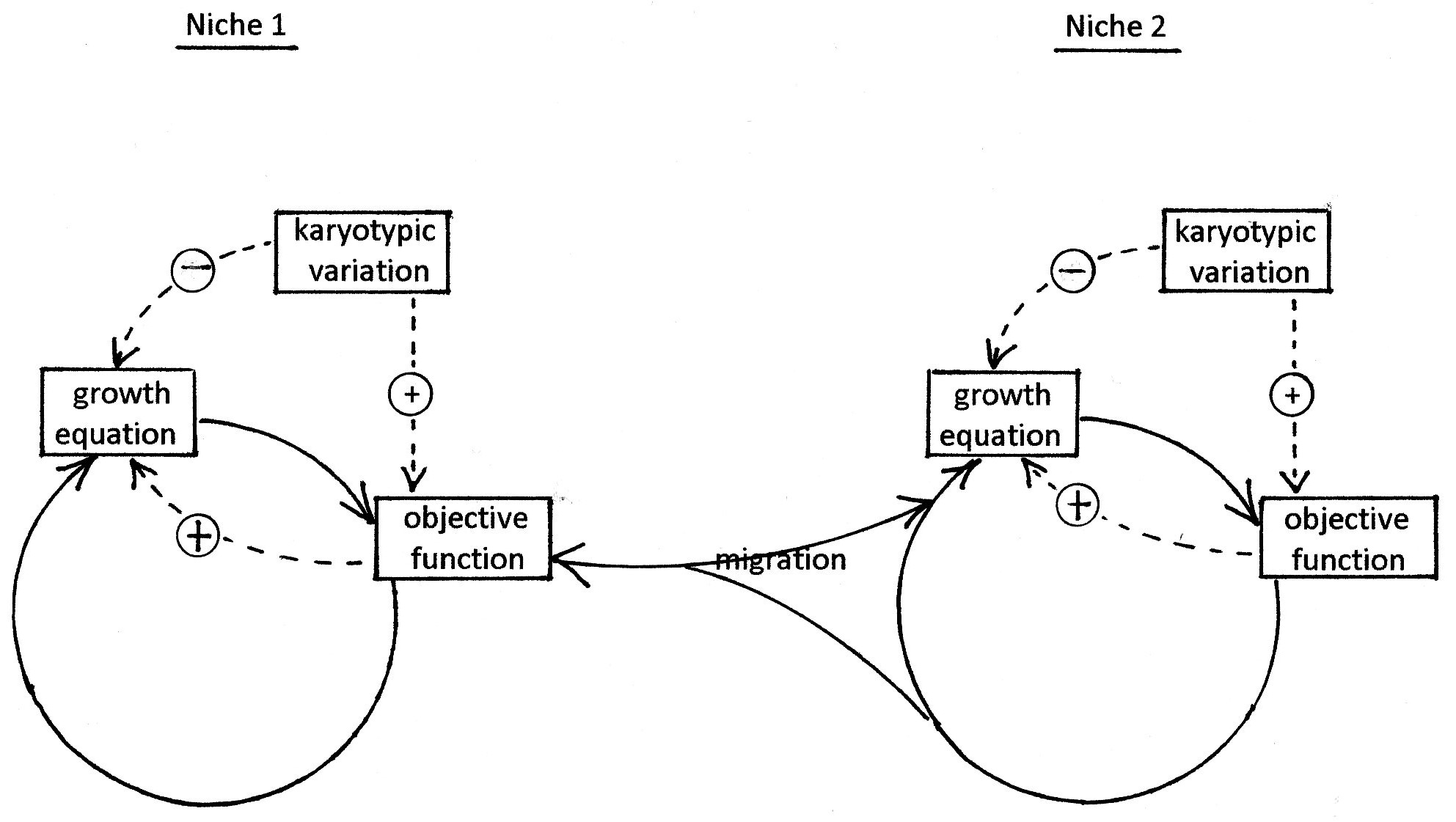
**

**Figure S1. The speciation algorithm.**

**Equations of the Sympatric Speciation Model**

In the following equations, populations may exist in one of two adjoining ecological niches.

The model describes six populations, np, described as zygotes, Z[np], which have a double set of chromosomes, or gametes, GA[np] which have a single set of chromosomes. Zygotes may be homozygous or heterozygous.

Z[1] are wild-type homozygotes in niche 1

Z[2] are heterozygotes in niche 1

Z[3] are rearranged homozygotes in niche 1

Z[4] are wild-type homozygotes in niche 2

Z[5] are heterozygotes in niche 2

Z[6] are rearranged homozygotes in niche 2

Z[0] is the total population in niche 1 = Z[1] + Z[2] + Z[3] equn. 1

Z[7] is the total population in niche 2 = Z[4] + Z[5] + Z[6] equn. 2

The proportion of wild-type homozygotes in niche 1, frac1 = Z[1] / Z[0] equn. 3

The proportion of heterozygotes in niche 1, frac2 = Z[2] / Z[0] equn. 4

The proportion of rearranged homozygotes in niche 1, frac3 = Z[3] / Z[0] equn. 5

The proportion of wild-type homozygotes in niche 2, frac4 = Z[4] / Z[7] equn. 6

The proportion of heterozygotes in niche 2, frac5 = Z[5] / Z[7] equn. 7

The proportion of rearranged homozygotes in niche 2, frac6 = Z[6] / Z[7] equn. 8

Gametes are formed once in each generation by meiosis:

GA[1] are wild-type gametes in niche 1 = 2 * Z[1] + Z[2] equn. 9

GA[2] are rearranged gametes in niche 1 = Z[2] + 2 * Z[3] equn.10

GA[4] are wild-type gametes in niche 2 = 2 * Z[4] + Z[5] equn.11

GA[5] are rearranged gametes in niche 2 = Z[5] + 2 * Z[6] equn.12

GA[0] is the total number of gametes in niche 1 = GA[1] + GA[2] equn.13

GA[7] is the total number of gametes in niche 2 = GA[4] + GA[5] equn.14

**Reproduction** falls into three stages: mitosis of germ cells (increase by cell division), meiosis (gamete formation) and fertilization (fusion of male and female gametes to form a next-generation zygote).

In each time increment, dt, the amount of mitosis in each population is given by the multiplication factor, fac[np].

fac[np]=exp(dt*log(2)/DT[np]) equn.15

where DT[np] are the population doubling times for population np.

If, because of chromosomal rearrangements, the fertility of gametes is decreased, as deduced from the spindle checkpoint dynamics, this results in lower population doubling time for heterozygotes, though the doubling time for rearranged homozygotes is unchanged. Thus if DT[1] =10, and the fertility of the rearranged population is halved, DT[2] = DT[1] *2 and DT[3] = DT[1]. Similarly, DT[5] = DT[4] * 2 and DT[6] = DT[4].

Then at each time interval, Z[np] *= fac[np] equn.16

Not all the members of a population survive to reproduce. The fraction of the population that dies before it can reproduce is termed the loss factor. The value of the loss factor depends on the growth curve being modelled. If exponential growth is modelled, cell loss per time incremant,

LF[np] = 0 until the carrying capacity of the niche is reached, after which LF[np] = 1 * dt.

equn.17

In the model, AP is the carrying capacity for niche 1, and AQ is carrying capacity for niche 2.

If Gompertzian growth is being modelled, the rate of death is proportionate to the difference between the total population and the carrying capacity, on a log scale:

LF[1] = Z[1] * log(Z[0])/log(AP) * (fac[1] -1) equn.18

LF[2] = Z[2] * log(Z[0])/log(AP) * (fac[2] -1) equn.19

LF[3] = Z[3] * log(Z[0])/log(AP) * (fac[3] -1) equn.20

LF[4] = Z[4] * log(Z[7])/log(AQ) * (fac[4] -1) equn.21

LF[5] = Z[5] * log(Z[7])/log(AQ) * (fac[5] -1) equn.22

LF[6] = Z[6] * log(Z[7])/log(AQ) * (fac[6] -1) equn.23

If logistic growth is being modelled, the rate of death caused by resource depletion is proportional to the difference between the total population and the carrying capacity, on a linear scale:

wabr = (Z[1]*fac[1] + Z[2]*fac[2] + Z[3]*fac[3]) / AP equn.24

(weighted average birthrate for niche 1)

wabq= (Z[4]*fac[4] + Z[5]*fac[5] + Z[6]*fac[6]) / AQ equn.25

(weighted average birthrate for niche 2)

Then for np=1 to np=3, Z[np] = Z[np] – Z[np]*wabr equn.26

for np=4 to np=6, Z[np] = Z[np] – Z[np]*wabq equn.27

**Fertilisation:**

The probability of a wild-type gamete being fertilised by another wild-type (or rearranged) gamete depends upon the proportion of the total gametes that are wild-type (or rearranged). The dynamics of fertilisation are unaffected by whether the chromosomal rearrangement is in the male or the female gamete.

Fraction of wild-type gametes in niche 1, gfrac1 = GA[1]/GA[0] equn.28

Fraction of rearranged gametes in niche 1, gfrac2 = GA[2]/GA[0] equn.29

Fraction of wild-type gametes in niche 2, gfrac3 = GA[4]/GA[7] equn.30

Fraction of rearranged gametes in niche 2, gfrac4 = GA[5]/GA[7] equn.31

In fertilization, two gametes fuse to form a single zygote, so

Z[1] = (GA[1] *gfrac1)/ 2 equn.32

Z[2] = (GA[1] * gfrac2 + GA[2] * gfrac1)/ 2 equn.33

Z[3] = (GA[2] * gfrac2)/ 2 equn.34

Z[4] = (GA[4] * gfrac3)/ 2 equn.35

Z[5] = (GA[4] * gfrac4 + GA[5] * gfrac3)/ 2 equn.36

Z[6] = (GA[5] * gfrac4)/ 2 equn.37

**Migration:**

The speciation algorithm assumes that the organism under study can survive in either of two ecological niches. These niches are overlapping or adjacent geographically, but differ in some factor that affects survival – temperature, water availability, availability of food or shelter, presence of predators or parasites. The original strain of the organism is poorly adapted to niche 2, and this is reflected in a higher death rate in this environment.

The initial assumption is that no organisms live in niche 2, but that when the carrying capacity of niche 1 is reached, organisms colonise niche 2 (e.g. in search of food). The migration may be one-way or two-way. Migration in each direction may be inversely proportional to food availability (i.e. directly proportional to population) or directly proportional to death rate.

Assuming one-way migration (colonization) from niche 1 to niche 2 occurs when the niche 1 population exceeds its carrying capacity:

Z[4] += XS * sf[1] * frac1 equn.38

Z[5] += XS * sf[2] * frac2 equn.39

Z[6] += XS * sf[3] * frac3 equn.40

where XS is the excess population in niche 1, i.e. Z[0] – AP equn.41

sf[np] are survival factors, a measure of the selective pressure in niche 2 compared with niche 1, e.g. if only one-tenth of the individuals that leave niche 1 make it to niche 2, then sf[1] = 0.1. The survival factors may mirror the death rates for individuals already in niche 2, but do not have to.

An alternative treatment of colonization assumes that the rate of migration is proportional to the selective pressure in niche 1, i.e. it gets greater as the total population gets closer to the carrying capacity:

Z[4] += Z[1] * Z[0] / AP * sf[1] equn.42

Z[5] += Z[2] * Z[0] / AP * sf[2] equn.43

Z[6] += Z[3] * Z[0] / AP * sf[3] equn.44

and reverse migration (niche 2 back to niche 1) is given by

Z[1] += Z[4] * Z[7] / AQ * sf[4] equn.45

Z[2] += Z[5] * Z[7] / AQ * sf[5] equn.46

Z[3] += Z[6] * Z[7] / AQ * sf[6]. equn.47

**Objective function**

If the total population in either niche exceeds the carrying capacity (i.e. if Z[0]>AP or Z[7]>AQ) the numbers of zygotes entering the next generation are recalculated so that the totals equal the carrying capacity, and the various sub-populations reflect the selective pressure resulting from their death rates:

Z[1] = AP * frac1 equn.48

Z[2] = AP * frac2 equn.49

Z[3] = AP * frac3 equn.50

Z[4] = AQ * frac4 equn.51

Z[5] = AQ * frac5 equn.52

Z[6] = AQ * frac6 equn.53

**The speciation algorithm:**

step 1: Set starting values for Z[1], Z[2], Z[3], Z[4], Z[5], Z[6]

step 2: Calculate reproduction factors for np=1 to np=6 (equation 15)

step3: Then for time = 0 to t

step 4: Calculate totals in niche 1 and niche 2 (equations 1 and 2).

step 5: Calculate fractional populations (equations 3 to 8).

step 6: For np = 0 to np = 6

calculate reproduction (equation 16)

calculate loss factors (equation 17, or equations 18 – 23 or equations 24 – 27, depending on the growth curve being used.

step 7: Calculate numbers of gametes (equations 9 – 12).

step 8: Calculate gamete totals in niche 1 and niche 2 (equations 13, 14).

step 9: Calculate gamete population fractions (equations 28 – 31).

step 10: Calculate new zygote formation resulting from fertilisation (equations 32 – 37)

step 11: Calculate colonization, if any (equation 41, then equations 38 – 40, or equations 42 – 47).

step 12: For np = 1 to 6, recalculate population fractions (equations 3 - 8)

step 13: If Z[0]>AP or Z[7]>AQ recalculate zygote populations from equations 48 – 53.

Loop back to step 3

**Table S1:** Effect of increasing death rate beyond the critical point

Speciation Algorithm version 1.02

----------------------------------------------------------

Time Z1 Z2 Z3 Ztotal

----------------------------------------------------------

0.0 10000.0 1.0 0.000 10001.0

400.0 4603.3 0.0 0.000 4603.3

800.0 2119.0 0.0 0.000 2119.0

1200.0 975.4 0.0 0.000 975.4

1600.0 449.0 0.0 0.000 449.0

2000.0 206.7 0.0 0.000 206.7

2400.0 95.1 0.0 0.000 95.1

2800.0 43.8 0.0 0.000 43.8

3200.0 20.2 0.0 0.000 20.2

3600.0 9.3 0.0 0.000 9.3

4000.0 4.3 0.0 0.000 4.3

4400.0 2.0 0.0 0.000 2.0

4800.0 0.9 0.0 0.000 0.9

5200.0 0.4 0.0 0.000 0.4

5600.0 0.2 0.0 0.000 0.2

6000.0 0.1 0.0 0.000 0.1

6400.0 0.0 0.0 0.000 0.0

6800.0 0.0 0.0 0.000 0.0

7200.0 0.0 0.0 0.000 0.0

7600.0 0.0 0.0 0.000 0.0

8000.0 0.0 0.0 0.000 0.0

----------------------------------------------------------

Doubling times: Z1 = 10.00 Z2 = 20.00 Z3 = 10.00

Death rates: Z1 = 0.070 Z2 = 0.070 Z3 = 0.070

Variants extinct at 19.0 w

**Table S2**: Increasing survival factor of Z1 entering niche 2 prevents allopatric speciation.

Speciation Algorithm version 2.12 Fri Feb 28 10:54:35 2020

-------------------------------------------------------------------------------

Time Z1 Z2 Z3 Ztotal Z4 Z5 Z6

-------------------------------------------------------------------------------

0.0 10000.0 1.0 0.000 10001.0 0.0 0.0 0.00

400.0 10342.5 0.0 0.000 10379.8 11696.4 0.4 0.00

800.0 10362.0 0.0 0.000 10399.5 11788.2 0.3 0.00

1200.0 10362.8 0.0 0.000 10400.3 11790.2 0.2 0.00

1600.0 10362.8 0.0 0.000 10400.3 11790.3 0.1 0.00

2000.0 10362.8 0.0 0.000 10400.3 11790.3 0.0 0.00

2400.0 10362.8 0.0 0.000 10400.3 11790.3 0.0 0.00

2800.0 10362.8 0.0 0.000 10400.3 11790.3 0.0 0.00

3200.0 10362.8 0.0 0.000 10400.3 11790.3 0.0 0.00

3600.0 10362.8 0.0 0.000 10400.3 11790.3 0.0 0.00

4000.0 10362.8 0.0 0.000 10400.3 11790.3 0.0 0.00

4400.0 10362.8 0.0 0.000 10400.3 11790.3 0.0 0.00

4800.0 10362.8 0.0 0.000 10400.3 11790.3 0.0 0.00

5200.0 10362.8 0.0 0.000 10400.3 11790.3 0.0 0.00

5600.0 10362.8 0.0 0.000 10400.3 11790.3 0.0 0.00

6000.0 10362.8 0.0 0.000 10400.3 11790.3 0.0 0.00

6400.0 10362.8 0.0 0.000 10400.3 11790.3 0.0 0.00

6800.0 10362.8 0.0 0.000 10400.3 11790.3 0.0 0.00

7200.0 10362.8 0.0 0.000 10400.3 11790.3 0.0 0.00

7600.0 10362.8 0.0 0.000 10400.3 11790.3 0.0 0.00

8000.0 10362.8 0.0 0.000 10400.3 11790.3 0.0 0.00

-------------------------------------------------------------------------------

Doubling times: Z1= 10.00 Z2= 20.00 Z3= 10.00 Z4=10.00 Z5=20.00 Z6=10.00

Death rates: Z1= 0.060 Z2= 0.060 Z3= 0.060 Z4=0.066 Z5=0.033 Z6=0.033

Survival factors: sf1=8.000e-002 sf2=1.000e+000 sf3=1.000e+000

sf4=0.000e+000 sf5=0.000e+000 sf6=0.000e+000

Variants extinct in niche 1 at 21.0 w
